# Multicentre evaluation of Xpert MTB/RIF assay in detecting urinary tract tuberculosis with urine samples

**DOI:** 10.1038/s41598-019-47358-3

**Published:** 2019-07-30

**Authors:** Yu Chen, Peng Wu, Liang Fu, Yu-hong Liu, Yao Zhang, Yanping Zhao

**Affiliations:** 1 Shenyang Tenth People’s Hospital and Shenyang Chest Hospital, Department of Tuberculosis, Shenyang, Liaoning Province 110044 P.R. China; 2 School of Public Health, Li Ka Shing Faculty of Medicine, The University of Hong Kong, 1/F, Patrick Manson Building (North), 7 Sassoon Road, Pokfulam, Hong Kong, Special Administrative Region P.R. China; 3 Shenzhen Third People’s Hospital affiliated to Southern University of Science and Technology, the 2nd Pulmonary Diseases Department, Shenzhen, Guangdong Province 518112 P.R. China; 4 China TB Clinical Trial Consortium, Beijing, 101149 P.R. China; 5 Global Research Services of Family Health International 360, Beijing, 101149 P.R. China

**Keywords:** Risk factors, Urology, Urinary tract infection

## Abstract

Genitourinary tuberculosis (GUTB) accounts for up to 40% of extrapulmonary tuberculosis cases. Rapid tests for GUTB are urgently needed because it is often associated with delayed health-care seeking, leading to serious consequences. This study evaluated the performance of the Xpert MTB/RIF assay in the rapid diagnosis of urinary tract tuberculosis (UTB) and rifampicin-resistant tuberculosis with urine specimens. In all, 302 patients were included from four hospitals in China. Suspected UTB patients were tested with Xpert, smear, and MGIT 960 culture. Drug susceptibility testing (DST) was conducted for culture-positive cases. The performance of the assays was evaluated against MGIT 960 culture and a composite reference standard (CRS). Among all participants, 150 (49.7%) had CRS-positive UTB, of whom 36 (24.0%) were culture-confirmed. Against culture, Xpert and smear achieved a sensitivity of 94.4% (95% CI: 81.3–99.3%) and 22.2% (95% CI: 10.1–39.2%), respectively. Against CRS, the sensitivity of Xpert, smear and culture was 41.3% (95% CI: 33.4–49.7%), 7.3% (95% CI: 3.7–12.7%), and 24.0% (95% CI: 17.4–31.6%). Xpert had better performance than smear and culture in detecting UTB from urine samples and could be considered for the diagnosis of UTB. Moreover, Xpert showed better performance than MGIT 960-based DST using urine culture.

## Introduction

Tuberculosis (TB), caused by *Mycobacterium tuberculosis* (MTB), has surpassed HIV/AIDS as the number one cause of death among all infectious agents^1,2^. In 2017, it was estimated that 1.6 million lives were claimed by TB, and 10.0 million people developed TB disease. TB can be classified as pulmonary TB and extrapulmonary TB (EPTB), with the latter accounting for approximately 14% of the 6.4 million reported TB cases, according to the World Health Organization (WHO) in 2017^2^. Genitourinary tuberculosis (GUTB), one of the most common forms of EPTB, is responsible for 15% to 40% of EPTB cases^3–7^. EPTB is a serious health challenge in both less and more developed regions. In England and Wales, the absolute number and proportion of EPTB cases among all TB cases increased significantly from 48% (2,717 cases) to 53% (4,205 cases) between 1999 and 2006^8^. The proportion of EPTB cases further increased to 59.6% but decreased in absolute number to 3,362 cases in 2016^9^. In the European Union and European Economic Area, between 2002 and 2011, the proportion of EPTB cases among all TB cases increased from 16.4% to 22.4%^10^. Globally, GUTB is often the second most common form of EPTB, only next to lymph node involvement^3,7^. The disease is often associated with delayed health-care seeking, leading to serious consequences^11^, especially in developing countries; thus, rapid tests for GUTB are urgently needed^12^. Delayed diagnosis and treatment of GUTB can lead to unilateral nonfunctioning in kidney and renal failure, which could be fatal^3,6^.

China is heavily affected by TB, ranking second in the number of estimated cases, as well as the number of multidrug-resistant TB (MDR-TB)/rifampicin-resistant TB (RR-TB) cases^2^. In 2016 and 2017, 836,236 and 835,193 cases of pulmonary TB were reported in China, respectively^13,14^. Together with India and Indonesia, these three countries accounted for 44% of all TB cases worldwide in 2017. At the same time, China (13%), India (24%), and the Russian Federation (10%) reported 47% of all MDR/RR-TB cases globally^2^.

Significant improvements have been made in the diagnosis of TB since the WHO updated its recommendation to use the rapid test called the Xpert MTB/RIF assay (Cepheid, Sunnyvale, CA, USA, referred as “Xpert” hereafter) for not only pulmonary TB but also EPTB in adults and children^15^. The extrapulmonary specimens recommended by the WHO that can be tested with Xpert include cerebrospinal fluid, lymph nodes, and tissue specimens. Potential contributions of other extrapulmonary specimens to the diagnosis of TB were limited by a lack of sufficient data. More comparative research is required to validate Xpert in detecting urinary tract tuberculosis (UTB) from urine samples. The current practice in diagnosing EPTB is by both culture and a composite reference standard (CRS)^16,17^.

In this multicentre study, our primary objective was to evaluate the performance of Xpert on urine specimens for the rapid diagnosis of UTB against BACTEC MGIT 960 system liquid media (Becton Dickinson, Sparks, MD, USA, referred as “MGIT 960” hereafter) among an HIV-negative population. The second objective was to evaluate Xpert in testing rifampicin-resistant MTB against the gold standard, namely, the MGIT 960 culture system^18^.

## Methods

### Study population

A multicentre study was conducted from January 2016 to September 2017 in four hospitals in China, including Shenyang Chest Hospital (Shenyang, Liaoning Province) in northern China, Shanghai Public Health Clinical Centre (Shanghai) in eastern China, Wuhan Pulmonary Hospital (Wuhan, Hubei Province) in central China, and Shenzhen Third People’s Hospital (Shenzhen, Guangdong Province) in southern China. All centres were based in urban areas in first- or second-tier cities. Suspected patients who had symptoms suggestive of UTB or a urine abnormality were included in this study. Patients who were HIV positive or had previously received anti-TB treatment for over a month or had a history of surgery to remove a kidney destroyed by tuberculosis were excluded. It has been reported that patients who have undergone nephrectomy often have a negative urine culture^19^.

### The gold standard

The results of Xpert testing were first compared with those of the gold standard (culture)^6^, i.e., those of MGIT 960 culture-positive cases. MGIT 960 had demonstrated better sensitivity than solid culture and was equivalent to the BACTEC 460 system, the previous gold standard for MTB, which has been discontinued by the manufacturer^20–22^. Given that culture is suboptimal in detecting EPTB, a composite reference standard (CRS) was also used as a standard for comparison, as recommended in the literature in the absence of an ideal gold standard^16,17,23^. In brief, patients were categorized into four groups based on the CRS: confirmed UTB (defined as patients with a positive culture), probable UTB (defined as patients with a negative culture but with clinical symptoms indicating UTB and with positive cystoscopy biopsy, or radiological signs), possible UTB (defined as patients with a negative culture but with clinical symptoms indicating UTB and all tests negative/refusal to do a cystoscopy but responsive to anti-TB treatment, i.e., improved after 3 months of anti-TB treatment) and not UTB (defined as patients with none of the above indicators and improvement without anti-TB treatment)^16,24,25^.

### Sample processing

Each patient enrolled in this study provided one early-morning, mid-stream urine specimen (≥5 mL) for smear, MGIT 960 culture, and Xpert assay testing.

#### Microscopy testing

Direct smears with 0.1–0.2 mL of each urine sample were prepared and stained using the auramine staining procedure. The specimens were then examined by light-emitting diode (LED) microscopy. The smears were read and interpreted in accordance with the guidelines^26^.

#### MGIT 960 preparation and MTB testing

In all, 2 mL of the urine sample, together with 1–2 times the volume of 2% N-acetyl-L-cysteine NaOH-Na citrate, was vortexed for 20 seconds before it was incubated for 15 minutes (room temperature). Phosphate-buffered saline (PBS) buffer (pH = 6.8) was then added to a final volume of 45 mL and centrifuged at 3000 × g for 15 minutes at 4 °C. Afterward, the supernatant was discarded, and the pellet was re-suspended with 1.5 mL of PBS. Then, 0.5 mL of the final product was placed into MGIT 960 liquid culture tubes and incubated at 37 °C, and a negative result would be determined at 42 days. If the reading at 42 days was positive, 0.1 mL was stained by auramine O and observed by fluorescence microscopy with Ziehl-Neelsen staining. If acid-fast bacilli (AFB) were observed, the result was defined as positive. The absence of the detection of AFB after repeated testing with culture and staining was considered a negative result. If the tube remained negative for 42 days, this result was also defined as negative. All procedures were performed following the MGIT 960 manual^27^.

#### Drug susceptibility testing (DST)

MTB cases with a positive culture were tested using an MGIT 960 IR kit (Becton Dickinson) against rifampicin (RIF, 40 μg/mL), according to the MGIT 960 manufacturer’s instructions^27^.

#### Xpert testing

In all, 1 mL of the urine sample was mixed with 2 mL of Xpert sample reagent. Then, the mixture was incubated for 15 minutes (room temperature) before 2 mL were collected to be placed into the Xpert cartridge and loaded onto the testing system (Cepheid, Sunnyvale, CA). The system was then run, and the results of the presence of MTB and RIF resistance were automatically generated after approximately 2 hours.

### Ethics statement

This study was approved by the Ethical Committee of the leading hospital, Shenyang Chest Hospital (No. 2017-007-02). As only routine samples were used in this study, the requirement for individual informed consent was waived by the review board. Following the policy issued by the General Office of the Central Committee of the Communist Party of China and the General Office of the State Council on *Deepening the Reform of the Evaluation and Approval Systems and Encouraging Innovation on Drugs and Medical Devices*, after ethical approval was obtained by the ethics committee of the clinical trial leader’s institute for this multi-centre study, all other authors recognized and accepted the conclusions of the ethics committee of the leading unit.

### Statistical analysis

Statistical analysis was performed using Stata 14.1 (Stata Corp, College Station, TX, USA). Chi-square test was used for the statistical comparison of categorical variables. A *P*-value < 0.05 was considered statistically significant. The parameters of Xpert and AFB were compared with those of the gold standard (culture) and the CRS-defined positive cases in terms of sensitivity, specificity, positive predictive value (PPV) and negative predictive value (NPV). The variables related to UTB detection (using CRS-defined cases) were analysed using logistic regression to generate the odds ratio (OR). Those with a *P*-value < 0.05 were then entered into the multivariable analysis to identify the independent risk factor(s).

## Results

### Study population

During the study period, a total of 327 patients with suspected UTB symptoms or a urine abnormality on routine testing were consecutively recruited. In all, 25 patients were excluded, including six HIV-positive patients and 19 post-nephrectomy patients due to TB. Finally, 302 patients with symptoms suggestive of UTB (181) or a routine urine test abnormality (121) were included, including 78 in Shenyang, 57 in Shanghai, 58 in Wuhan and 109 in Shenzhen.

**Figure 1:**
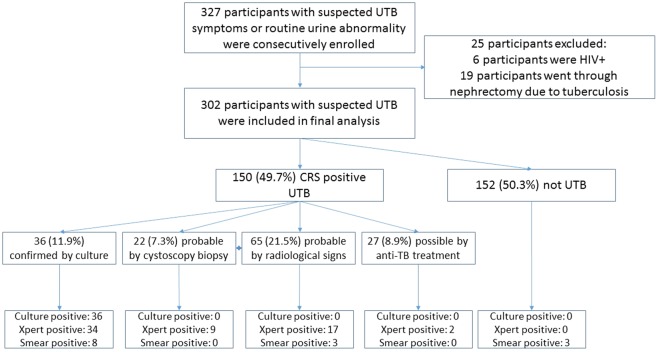
The flow of patient inclusion (all percentages report the proportion of 302 participants).

The flow of patient inclusion is shown in Fig. 1. A total of 327 eligible participants were consecutively enrolled. After screening the exclusion criteria, 302 participants were left in the final analysis, including 150 (49.7%) considered to have UTB based on CRS, and 152 (50.3%) without UTB. Of the 150 CRS-defined cases of UTB, 36 (24.0%) were confirmed by culture, 87 (58.0%) were probable UTB, including 22 (14.7%) by cystoscopy biopsy and 65 (43.3%) by radiological signs, and 27 (18.0%) were possible UTB according to the CRS definition presented in the methods.

### Performance analysis of Xpert assay for UTB detection

The performance of the three assays, namely, Xpert, AFB and MGIT 960, is shown in Table 1 below.

**Table 1:** Sensitivity, specificity, and positive and negative predictive values of Xpert, AFB smear and MGIT 960 in detecting UTB from urine specimens.

Gold standard	Screening method	Test performance			
		Sensitivity (95% CI)	Specificity (95% CI)	PPV (95% CI)	NPV (95% CI)
MGIT 960 culture	Xpert	94.4% (34/36)	89.5% (238/266)	54.8% (34/62)	99.2% (238/240)
		(81.3–99.3%)	(85.1–92.9%)	(41.7–67.5%)	(97.0–99.9%)
	AFB smear	22.2% (8/36)	97.7% (260/266)	57.1% (8/14)	90.3% (260/288)
		(10.1–39.2%)	(95.2–99.2%)	(28.9–82.3%)	(86.3–93.4%)
CRS	Xpert	41.3% (62/150)	100% (152/152)	100% (62/62)	63.3% (152/240)
		(33.4–49.7%)	—	—	(56.9–69.4%)
	AFB smear	7.3% (11/150)	98.0% (149/152)	78.6% (11/14)	51.7% (149/288)
		(3.7–12.7%)	(94.3–99.6%)	(49.2–95.3%)	(45.8–57.6%)
	MGIT 960	24.0% (36/150)	100% (152/152)	100% (36/36)	57.1% (152/266)
		(17.4–31.6%)	—	—	(51.0–63.2%)

CI, confidence interval; PPV, positive predictive value; NPV, negative predictive value.

Using MGIT 960 liquid culture as the gold standard, the sensitivity of Xpert was 94.4% (34/36), (95% CI: 81.3–99.3%), which exceeded that of AFB (*P* < 0.0001). The specificity of Xpert was 89.5% (238/266) (95% CI: 85.1–92.9%), which was lower than that of AFB, the specificity of which was 97.7% (260/266) (95% CI: 95.2–99.2%) (*P* < 0.0001).

Compared with the CRS, Xpert’s sensitivity was 41.3% (62/150) (95% CI: 33.4–49.7%), which was significantly better than that of AFB (*P* < 0.0001) and MGIT 960 (*P* < 0.01). The specificity of Xpert was 100.0%, which was the same as that of MGIT 960.

Using culture as the gold standard, the PPVs of Xpert and AFB were similar, at 54.8% (34/62) (95% CI: 41.7–67.5%) and 57.1% (8/14) (95% CI: 28.9–82.3%), while the NPV of Xpert was 99.2% (238/240) (95% CI: 97.0–99.9%), considerably better than that of AFB (*P* < 0.0001).

Using CRS as the reference, the PPVs of Xpert and MGIT 960 were both 100.0%. The NPV of Xpert was 63.3% (152/240) (95% CI: 56.9–69.4%), which was higher than that of MGIT 960, although the difference was not statistically significant.

As shown in Table 2, the sensitivity of Xpert was similar in all four sites of this study, with no significant differences among the sites. The sensitivities of MGIT 960 and AFB smear in the four centres were largely similar.

**Table 2:** Sensitivity of Xpert MTB/RIF, MGIT 960 and AFB smear in detecting UTB (based on CRS) in different cities.

Screening method	Shenyang (39) n (%)	Shenzhen (55) n (%)	Wuhan (30) n (%)	Shanghai (26) n (%)	Total (150) n (%)
MGIT 960 positive	9 (23.1)	12 (21.8)	8 (26.7)	7 (26.9)	36 (24.0)
Xpert positive	16 (41.0)	20 (36.4)	14 (46.7)	12 (46.2)	62 (41.3)
AFB smear positive	2 (5.1)	3 (5.5)	2 (6.7)	4 (15.3)	11 (7.3)

### Detection of RIF resistance

Out of the 150 UTB cases diagnosed by CRS, Xpert identified three cases resistant to RIF. MGIT 960 DST identified two cases from urine samples, missing one UTB case based on Xpert results. The only mismatched case was re-examined. In this case, the patient was hospitalized with sacroiliac joint tuberculosis symptoms, such as low back pain and walking difficulties. MGIT 960 was performed using a sacroiliac pus specimen and urine (due to a routine urine test abnormality). Though the urine sample culture was negative for MTB, the sacroiliac joint pus specimen was positive for MTB and RIF, indicating that this patient had RIF resistance. The sample was tested twice with culture and the Xpert assay using urine, and the results were consistent.

### Socio-demographic information and risk factors associated with UTB

The socio-demographic information of the 302 participants is shown in Table 3. The mean age of the participants was 53 years, ranging from 19 to 85 years, with females in a slight majority (55.3%). In all, 68.5% of the participants reported no history of TB.

**Table 3:** Demographic characteristics and association with UTB in the 302 study participants.

Characteristics	Diagnostic Class				
	UTB cases by CRS (150) n (%)	Non-UTB cases (152) n (%)	cORs (95% CI)	aORs (95% CI)	Total (302) N (%)
Female	70 (46.7)	97 (63.8)	1	1	167 (55.3)
Male	80 (53.3)	55 (36.2)	2.02 (1.27–3.20)**	1.92 (1.16–3.18)*	135 (44.7)
Age <30 years	5 (3.3)	11 (7.2)	1	1	16 (5.3)
30–60 years	90 (60.0)	106 (69.7)	1.87 (0.63–5.58)	1.22 (0.38–3.89)	196 (64.9)
>60 years	55 (36.7)	35 (23.0)	3.46 (1.11–10.8)*	1.90 (0.56–6.38)	90 (29.8)
Duration of illness <3 months	74 (49.3)	96 (63.2)	1	1	170 (56.3)
3–12 months	62 (41.3)	52 (34.2)	1.55 (0.96–2.49)^+^	1.20 (0.71–2.02)	114 (37.8)
>12 months	14 (9.3)	4 (2.6)	4.54 (1.44–14.4)*	1.26 (0.34–4.68)	18 (6.0)
Resident	73 (48.7)	93 (61.2)	1	1	166 (55.0)
Migrant	77 (51.3)	59 (38.8)	1.66 (1.05–2.63)*	1.36 (0.82–2.24)	136 (45.0)
TB history No	79 (52.7)	128 (84.2)	1	1	207 (68.5)
Yes	71 (47.3)	24 (15.8)	4.79 (2.79–8.23)**	4.22 (2.38–7.49)**	95 (31.5)

**P < 0.01, *P < 0.05, ^+^*P* < 0.1, OR, odds ratio, aOR, adjusted odds ratio.

The risk factors associated with being diagnosed with UTB (according to the CRS) were examined. In the univariate analysis, all of the key socio-demographical variables were significantly related to the outcome variable. Those who were older, male, from a rural area, had a history of TB, and with a longer duration of illness were more likely to be diagnosed with UTB. As these variables were all significant (*P* < 0.05) in the univariable analysis, they were all entered into the multivariable analysis. The multivariable analysis identified two independent variables significantly associated with being diagnosed with UTB, namely, a TB history and the male sex. Those who reported a history of TB were approximately four times as likely to be diagnosed with UTB than those without a history of TB, with an aOR = 4.22 (95% CI: 2.38–7.49). Males were approximately twice as likely to be diagnosed with UTB than females, with the aOR = 1.92 (95% CI: 1.16–3.18).

## Discussion

The Xpert MTB/RIF assay showed superior performance in terms of sensitivity against AFB smear when using BACTEC MGIT 960 system liquid media as the gold standard and against both AFB smear and MGIT 960 when CRS was used as the reference. These findings are in line with a previous systematic review and meta-analysis that identified Xpert as having good sensitivity and specificity^28^. The excellent performance of Xpert in identifying RIF resistance has also been demonstrated. In addition, key independent risk factors for UTB were explored.

The key strengths of this study include the geographical coverage (northern eastern, central, and southern China), the large sample size and the use of MGIT 960 system liquid media in comparison with Xpert in terms of performance. However, there are also several limitations. First, only one early-morning urine specimen was used for all the testing in this study, and it has previously been reported that the bacterium might be intermittently excreted, leading to false-negative results due to the use of a single sample^29^. In another paired study, centrifugation of the urine samples could increase the performance of Xpert^30^. However, early-morning urine specimens were chosen for use in this study according to its recommendation by the National Institute for Health and Care Excellence (NICE) as the ideal sample for testing GUTB with culture^31^. Future studies should, however, ascertain the optimal urine sample for the Xpert test. Second, due to the low prevalence of HIV among the general population in China, HIV-infected patients were excluded, which renders our conclusion potentially not applicable to HIV-infected individuals. Future studies with HIV-infected individuals could be considered.

Against culture, Xpert’s sensitivity was 94.4% (95% CI: 81.3–99.3%), which was very similar to that reported in a previous study by Pang *et al*., i.e., 94.6% (95% CI: 87.3–100.0%)^25^. However, Pang *et al*. used Löwenstein-Jensen (L-J) culture instead of liquid culture. It has been reported that liquid media-based culture has better performance than solid culture in terms of the isolation rate^20^. In other studies, MGIT 960 demonstrated better sensitivity than solid culture^21,22^. In fact, MGIT 960 was equivalent to the BACTEC 460 system, the gold standard for MTB (but discontinued by the manufacturer)^20–22^. The MGIT 960 culture system was also the gold standard for RIF-resistant MTB^18^. The sensitivity identified in this study was lower than that reported in previous research by Hillemann *et al*., i.e., 100.0%, but the results should be read with caution as only 6 positive cases were identified by Xpert among 91 urine samples^32^. Another study in Italy reported a sensitivity and specificity for Xpert of 92.3% and 99.0% respectively, with 15 positive cases identified by Xpert (out of 130 urine samples)^24^. In comparison, our study had a much larger sample size and therefore a higher statistical power for the findings presented. Interestingly, against the CRS, the sensitivity of Xpert in our study was 41.3% (95% CI: 33.4–49.7%), which was lower than that reported by Pang *et al*., at 63.0% (95% CI: 52.4–73.5%); in addition, the sensitivity of culture was also lower in our study, at 24% (95% CI: 17.4–31.6%), than in Pang *et al*.’s study, at 45.7% (95% CI: 34.8–56.5%)^25^. The difference could be due to the distinct urine samples used, in that the samples used in Beijing could generate a better outcome than those used in the four centres included in this study. However, the findings of the four centres in our study were consistent and could therefore be more representative. Similar to another study^25^, Xpert detected all the RIF-resistant isolates, with even better performance in identifying RIF-resistance using urine samples. However, as there are very few isolates with resistance in the accessible studies (five in Pang *et al*.’s study and three in our study), further studies are needed to validate Xpert’s performance in the detection of RIF resistance.

Our study indicates that Xpert could be used as an assay for the diagnosis of UTB, partially addressing the research questions set by the WHO, based on a retrospective evaluation of the performance of Xpert in identifying both MTB and RIF resistance using urine samples^15^. Future studies should involve a financial assessment of Xpert testing to fully evaluate the cost-effectiveness of using Xpert to avoid a delayed diagnosis of UTB and the resulting serious consequences. The key independent risk factors identified are a history of TB and the male sex, which could also be put into an algorithm to evaluate the benefit of the targeted vs non-targeted screening of all potential patients. The performance of Xpert in testing different urine samples, namely, early-morning urine samples, 24-hour urine sediment samples, and concentrated samples from multiple days, should be evaluated in order to identify the optimal sample for diagnosing UTB by Xpert.

## Data Availability

The datasets generated and analysed in the current study are available from the corresponding author on reasonable request.
